# 原发性肺淋巴瘤1例

**DOI:** 10.3779/j.issn.1009-3419.2014.10.10

**Published:** 2014-10-20

**Authors:** 青春 赵, 森 韦, 昕 李, 清华 周, 军 陈

**Affiliations:** 300052 天津，天津医科大学总医院肺部肿瘤外科 Department of Lung Cancer Surgery, Tianjin Medical University General Hospital, Tianjin 300052, China

**Keywords:** 肺肿瘤, 原发性肺淋巴瘤, 诊断, 治疗, Lung neoplasms, Primary pulmonary lymphoma, Diagnosis, Treatment

## Abstract

原发性肺淋巴瘤（primary pulmonary lymphoma, PPL）是一种起源于淋巴结外，累及肺实质和（或）支气管的克隆性淋巴细胞增殖性疾病。PPL临床非常罕见，仅占肺部原发性恶性肿瘤的0.5%-1%，常因其临床特征及影像学特征无特异性而容易被误诊或漏诊。该病的治疗存在争议，手术及术后的辅助化疗为目前多数患者的治疗方案。本文将报道1例罕见的原发性肺淋巴瘤，并初步讨论其诊断和治疗。

## Abstract (en)

Primary pulmonary lymphoma (PPL) is an extranodally original disorder, which is involving the lung parenchyma and (or) bronchial with clonal lymph cell abnormal proliferation. PPL is very rare in clinics, accounting for only 0.5%-1% of primary lung tumors, and can easily be misdiagnosed or missed due to the nonspecifically clinical features and imaging findings. Although the treatment of this disease has controversial, surgery and postoperative adjuvant chemotherapy are used for most patients in present. This review will report a rare case of primary pulmonary lymphoma, and discuss its diagnosis and treatment.

## 1 临床资料

患者，女，32岁，于2012年11月主因“咳嗽、咳痰伴痰中带血”于外院就诊，初步诊断为肺部炎症，给予抗炎治疗1周后（具体诊疗经过不详），症状未见明显好转就诊于其他医院。在其住院期间行痰脱落细胞学检查，可见异型细胞，考虑为癌细胞。气管镜检查未见明显异常。胸部强化计算机断层扫描（computed tomography, CT）检查提示：右肺上叶纵隔旁肿物，内部可见含气支气管征，肿物周围大量磨玻璃密度影（图 1A、1B），考虑肺癌可能性大。在外院给予GP（顺铂+吉西他滨）方案化疗5周期。于第2次化疗周期结束后复查胸部CT，提示肿物体积较化疗前略缩小（图 1C）。患者临床咳嗽症状未见明显改善，但已无痰中带血。第5次化疗周期结束后再次复查胸部CT，示肿物体积较第2次化疗后明显增大（图 1D），且化疗期间再次出现痰中带血症状。患者为求进一步治疗，遂来我院就诊，以“肺部肿物”入院。入院查体一般情况可，未见明显异常，特别是全身浅表淋巴结无肿大。实验室检查未见异常，上腹部强化CT、全身骨扫描和头颅增强核磁均未见明显异常。完善术前检查后于2013年8月21日全麻下行“右肺上叶切除加系统淋巴结清扫术”。术中见肿瘤位于纵隔旁右肺上叶内，脏层胸膜皱缩，大小约4 cm×3.5 cm×2 cm，呈暗灰白色，纵隔淋巴结未见肿大。如图 2所示，术后病理报告为右肺上叶弥漫性大B细胞淋巴瘤，非GCB型。免疫组化检测示CD20、CD79a、PAX5、Bcl-6和Mum-1的蛋白表达均为阳性，其中，Ki-67在80%的肿瘤细胞中表达阳性；而CK、EMA、CD3、CD10、CD56、CD68和S-100的蛋白表达却均为阴性。支气管断端未见肿瘤细胞累及，支气管旁淋巴结及第2、3、4、7、9组淋巴结未见肿瘤累及。患者术后恢复顺利，病情好转出院。目前在当地医院肿瘤内科继续治疗。

**1:**
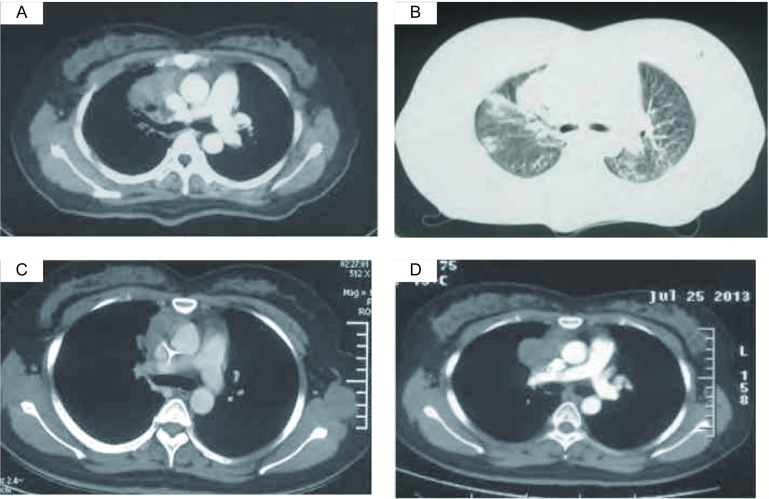
不同时间段的患者胸部CT检查。A-B：化疗前患者的胸部CT（2012-12-4），示右肺上叶纵隔旁肿物，内部可见含气支气管征，肿物周围大量磨玻璃密度影；C：第2次化疗周期结束后的胸部CT（2013-1-22），示肿物体积较化疗前略缩小；D：第5次化疗周期结束后的胸部CT（2013-7-25），示肿物体积较第2次化疗结束后明显增大。 CT scans of the chest during different times. A-B: The chest CT before chemotherapy (2012-12-4), indicating the mass in the upper lobe of the right lung. The mass was closed to the mediastinum and contained air brochogram. There were amount of ground glass density shadows around the mass; C: The chest CT scan after the second cycle of chemotherapy (2013-1-22). The volume of the mass was slightly decreased compared to the beginning; D: The chest CT scan after the fifth chemotherapy (2013-7-25). The volume of the mass was increased compared to the mass after the second chemotherapy. CT: computed tomography.

**2:**
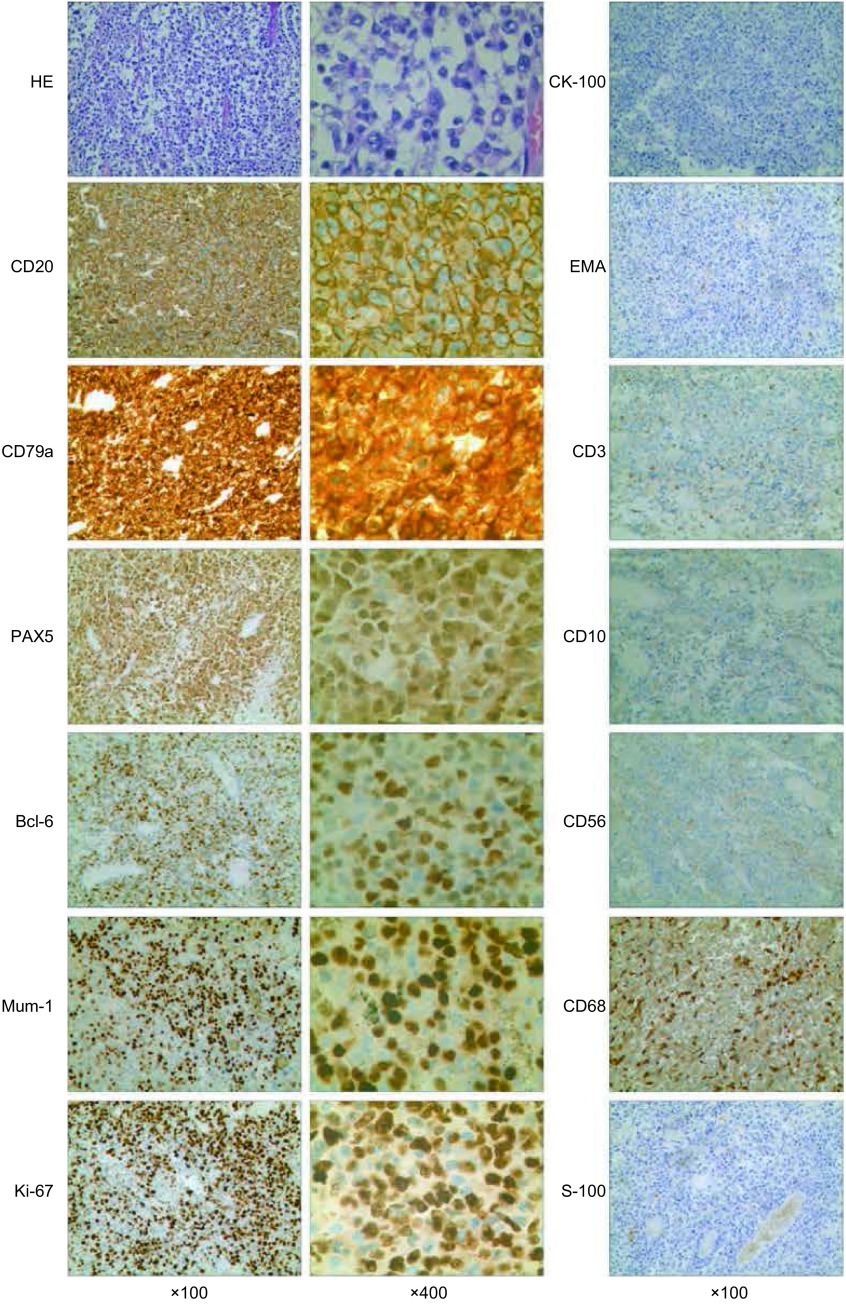
组织标本的病理分析。HE染色证实为（右肺上叶）弥漫性大B细胞淋巴瘤，非GCB型。免疫组化染色显示CD20、CD79a、PAX5、Bcl-6和Mum-1蛋白表达均为阳性，Ki-67蛋白表达80%细胞染色为阳性；而CK、EMA、CD3、CD10、CD56、CD68和S-100的蛋白表达均为阴性。 The analysis of the pathology. HE staining indicated the diffuse large B cell lymphoma (None GCB); The immunohistochemistry staining proved that the protein expressions of CD20, CD79a, PAX5, Bcl-6 and Mum-1 are positive, as well as the 80% of tumor cells staining positive with anti-Ki-67 antibody; Meanwhile, the expression of CK-100, EMA, CD3, CD10, CD56, CD68 and S-100 were negative by immunohistochemistry detection.

## 2 讨论

原发性肺淋巴瘤（primary pulmonary lymphoma, PPL）是指起源于淋巴结外，累及肺实质和（或）支气管的克隆性淋巴细胞增殖性疾病。病理学上分为霍杰金淋巴瘤和非霍杰金淋巴瘤两大类，大多数为非霍杰金淋巴瘤，且多数为黏膜相关淋巴组织淋巴瘤，以B细胞淋巴瘤为主。本例患者病理为弥漫性大B细胞淋巴瘤，非GCB型，属于非霍杰金淋巴瘤的一种。原发性肺淋巴瘤临床非常罕见，仅占肺部原发性恶性肿瘤的0.5%-1%^[1]^。发病高峰年龄为50岁-60岁，30岁以下少见，女性多于男性。本病多起病隐匿，呼吸道症状较轻，部分患者可有咳嗽、咳痰、胸痛等非特异性表现，偶有患者出现咯血、呼吸困难等症状。胸部CT可分为炎症型和结节肿块型。前者表现为单发或多发弥漫性斑片状模糊影，毛玻璃样影和实变影，内可含支气管充气征；后者表现为单发或多发的结节或肿块，周围型多见，呈类圆形，边界清楚或模糊，可有分叶及毛刺^[2]^。由于本病较少见，且在影像学上很难与肺癌区分，加之无特异性临床表现，故很难与肺癌鉴别，容易误诊或漏诊，因此对于临床表现较轻，肺部病灶生长缓慢的患者，应考虑到PPL的可能。其最终确诊还是要取决于病理组织学的证据，可依靠纤支镜肺活检或经皮肺穿刺活检取得病理。

许多学者^[3, 4]^认为手术是治疗PPL的主要方法，尤其是一些局限性的病例，手术既有明确诊断的作用，又能达到治疗的目的，同时也为下一步放、化疗提供依据。本例患者术前病理诊断不明确，拟诊为肺癌后按照肺癌化疗方案治疗未见明显获益，且病程中反复出现咯血症状，威胁生命，故选择手术治疗为最佳治疗方案。

该病是一种预后相对较好的恶性肿瘤，其预后因肿瘤的病理组织学类型、分期以及手术后是否进行正规的放化疗而异。5年生存率67%-93%，中位生存时间大于10年^[5, 6]^。目前多采用放疗和化疗为主的综合保守治疗方案^[7]^。化疗药物国内多选择标准的CHOP（环磷酰胺+多柔比星+长春新碱+泼尼松）方案或者R-CHOP（利妥昔单克隆抗体+CHOP）方案。近年来有相关报道^[8]^称利妥昔单克隆抗体联合CHOP方案治疗CD20阳性的DLBCL与单纯CHOP方案相比，能明显提高疗效，同时并不增加化疗的毒副反应。对于手术确诊的患者，建议其术后继续辅助化疗。手术治疗结合化疗或者单纯化疗，疗效均较满意。
